# Assessing community health workers’ level of knowledge concerning their roles in water, hygiene, and sanitation in Comè-Bopa-Grand Popo-Houéyogbé, Benin (2024)

**DOI:** 10.3389/fpubh.2025.1646722

**Published:** 2025-10-14

**Authors:** Parfait Wouékpé, Cyriaque Dégbey, Alphonse Kpozéhouen

**Affiliations:** ^1^Doctoral School of Health Sciences, Faculty of Health Sciences, University of Abomey-Calavi, Abomey-Calavi, Benin; ^2^Department of Health and Environment, Regional Institute of Public Health, University of Abomey-Calavi, Abomey-Calavi, Benin; ^3^Department of Epidemiology and Biostatistics, Regional Institute of Public Health, University of Abomey-Calavi, Abomey-Calavi, Benin

**Keywords:** community health workers, WASH, knowledge, supervision, training, Benin

## Abstract

**Objectives:**

This study assessed the level of knowledge of community health workers (CHWs) regarding water, hygiene, and sanitation (WASH) in the Comè-Bopa-Grand Popo-Houéyogbé health zone in Benin.

**Methods:**

A cross-sectional survey was conducted among 160 CHWs selected randomly. Data were collected using a structured questionnaire and analyzed with SPSS 21.0. Logistic regression was used to identify factors associated with CHW knowledge.

**Results:**

Most CHWs (68.8%) had insufficient knowledge of their roles in WASH. Factors significantly associated with good knowledge included Mina ethnicity (OR = 0.3; 95% CI: 0.1–0.9), being married (OR = 10.0; 95% CI: 1.3–77.7), training on activity packages (OR = 3.3; 95% CI: 1.7–10.0), supervision by a qualified agent (OR = 10.2; 95% CI: 2.5–40.6), and participation in group follow-up sessions (OR = 10.0; 95% CI: 5.0–48.9). Multivariate analysis showed that attending at least two group sessions greatly increased the likelihood of good knowledge (OR = 23.9; 95% CI: 5.3–107.7).

**Conclusion:**

Strengthening CHW training, regular follow-up, and incentives is essential to improve WASH-related knowledge and public health impact.

## Introduction

Access to quality water, proper hygiene and adequate sanitation is a major public health issue, particularly in developing countries. Several studies show that waterborne diseases, such as diarrhea and cholera, represent a significant cause of morbidity and mortality (1). According to the World Health Organization (WHO) (2), infections linked to contaminated water continue to affect millions of people each year, particularly in regions with inadequate sanitation infrastructure. In Benin, reports from the Ministry of Health indicate that a significant proportion of the population is exposed to health risks due to limited access to drinking water, with a national rural service rate estimated at 79.4% in December 2023, and optimal sanitation conditions (3).

In this context, community health workers, these field actors who act as a link between health authorities and the population, play a key role in disseminating good hygiene and sanitation practices (4). The community health worker is any person, whether a member of the community or not, who has received training to deal with the health problems of individuals and the community and to work in close collaboration with human, animal and environmental health services, local elected officials, the local component of the health system (CoLoSS) and other actors involved in community health, without necessarily being a health professional. He has a threefold mission: (i) to bring health services to the very place where people live and work; (ii) to help communities recognize their own health needs; (iii) to help the population solve their own health problems. He is therefore responsible for ensuring health promotion through information, education and communication on the one hand and, on the other hand, the management of a case (non-drug, without invasive procedures), its referral and follow-up. To do this, he will carry out home visits and focus groups (5, 6).

However, while global statistics on the prevalence of conditions related to poor water quality are available, it is clear that the specific knowledge of community health workers regarding their roles and responsibilities remains poorly documented. Indeed, current literature often focuses on global health indicators without exploring in depth how these local actors perceive and implement guidelines relating to hygiene promotion and sanitation (7, 8). This gap is particularly worrying in specific areas such as Comè-Bopa-Grand Popo-Houéyogbé, where problems related to water quality and sanitation conditions are exacerbated by fragile socio-economic contexts with telling epidemiological data such as the most frequent ailments received in consultations, malaria (47%), acute respiratory infections (16.8%), diarrhea and other gastrointestinal ailments (7%) and at the level of children under 5 years these data are more pronounced acute respiratory infections (28.3%), diarrhea and other gastrointestinal ailments (8.7%) (9).

These health indicators highlight the magnitude of water and sanitation-related challenges in the study area. However, they do not provide information on how community health workers (CHWs), who are the frontline actors for health promotion, understand and implement their roles. If CHWs have limited or inaccurate knowledge, their capacity to influence household practices is reduced, which may partly explain the persistence of preventable diseases. Therefore, assessing their level of knowledge is essential to better understand their effective contribution to the observed health outcomes.

The lack of detailed data on the training, understanding and application of responsibilities by these relays raises questions about the effectiveness of public health interventions in the region.

In view of these findings, this work aims to study the level of knowledge of community health workers on their roles and responsibilities in water quality, hygiene and sanitation in the Comè-Bopa-Grand Popo-Houéyogbé health zone.

## Methods

### Setting

**Figure 1:**
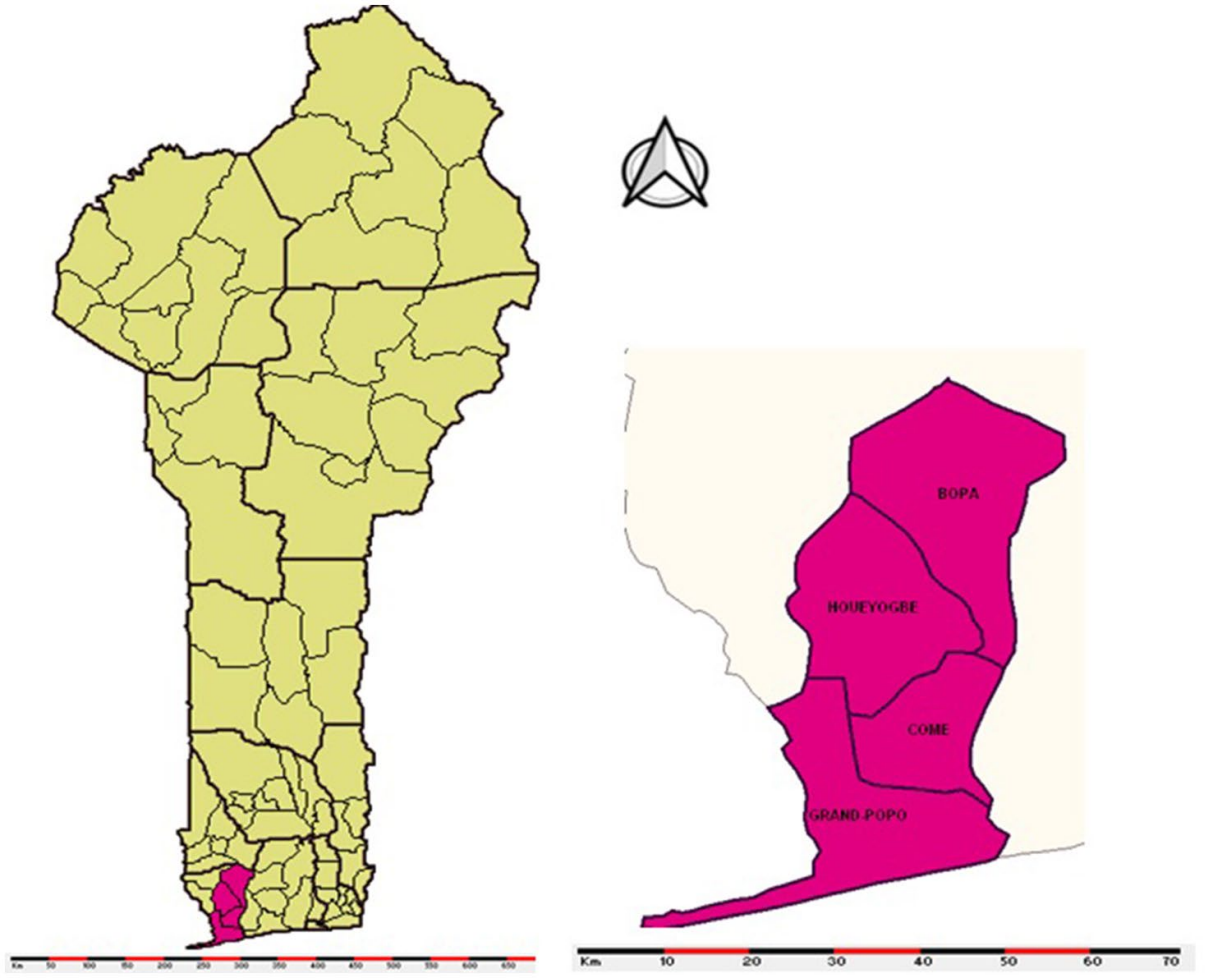
Geographic map of the Comè Bopa Grand Popo Houéyogbé health zone, 2024.

This study took place in the health zone of Comè, Bopa, Grand-Popo and Houéyogbé. It covers an area of 1,120 km^2^ and is located in the Mono department of Benin (10). The health zone has three large lakes: Lake Ahémé, Lake Toho and Lake Togbadji. The main rivers are: the Mono, the Couffo and the Sazué (11) (Figure 1).

### Study design

This was a descriptive and analytical cross-sectional study aimed at assessing the level of knowledge of community health workers regarding their roles and responsibilities in maintaining water quality, hygiene and sanitation. This investigation, conducted in the field, involved three target groups: community health workers, households in the villages covered by these relays and health workers (including post chiefs). Data was collected from September 18 to October 15, 2024.

### Participants

To be included in the study, participants were required to meet the following criteria: be a community health worker, a member of a household in a village served by a relay, or a health worker present at the post, and have resided in the area for at least 1 year. Conversely, new community health workers (with less than 1 year of experience), health workers on leave, sick or absent during the survey, as well as people residing in the area for less than 1 year were excluded.

This was a two-stage sampling. In the first stage, the most populated districts were selected in each stratum (each commune in the health zone) proportionally to the size of the population. In the second stage, the villages were selected in the selected districts randomly based on their list (approximately 40 villages per district). Then, in each selected village, the relay was systematically counted. The sample size was calculated by applying the Schwartz formula $n=Z_{\alpha }^{2}\frac{\mathrm{pq}}{i^{2}}$ where *n* is the size of the sample drawn, *p* is the theoretical percentage of good knowledge among the RC (*p* = 0,0375), *q (1-p)* is the theoretical percentage of poor knowledge, Z _𝜶_ is the Z score for the confidence level (95%) (here Z _𝜶_ =1.96) and *i* the margin of error (*i =* 5%). This calculation made it possible to estimate a minimum size of 154 relays, adjusted to 160 to ensure a fair distribution (i.e., approximately 40 relays per municipality in the Health Zone).

### Data sources/measurement

Data collection took place from September 18 to October 15, 2024, and was carried out primarily through direct interviews using a structured questionnaire administered to community health workers, households, and health workers. Field observations supplemented these interviews to adequately assess the population’s commitment to promoting a healthy environment.

### Variables

#### Dependent variable

The dependent variable of the study was the level of knowledge of community health workers about their roles and responsibilities. This is the percentage of activities to be carried out notified by the RC. Thus, it was a dichotomous variable with the modalities:

- Good knowledge: a percentage of satisfactory answers greater than 50%
- Insufficient knowledge: A percentage of satisfactory answers less than or equal to 50%

#### Independent variables

Explanatory variables included:

- Factors: age, gender, level of education, religion, ethnicity, marital status, occupation, place of residence;
- Factors related to the community health workers task: seniority of at least 5 years, possession of an electronic device (smartphone or tablet), training on the activity packages to be carried out, training on hygiene and sanitation, monitoring by a community agent, the number of group monitoring sessions and continuing training;
- Factors: Free consultation at the CS, official presentation of the community health workers to the locality, benefits or bonuses, involvement in mass vaccination campaigns.

### Statistical methods

Kobocollect software and data cleaning with SPSS version 21.0 software, we performed all statistical analyses using the same software. The chi-square test was used to assess the relationship between the level of knowledge of community relays and the explanatory variables. Bivariate and multiple logistic regression models were used to examine the probability of the level of knowledge of community relays as a function of the explanatory variables. The *p*-values obtained from the bivariate regression analysis were used to decide on the inclusion of variables in the multivariate model. Variables with a *p*-value less than 0.20 were included in the multivariate model. All independent variables that met the selection criterion (*p* < 0.20) were included in the final model, taking into account potential confounders. The association between the level of knowledge of community health workers and the explanatory variables was assessed by the odds ratio (OR) followed by their 95% confidence interval (95% CI).

## Results

### Sociodemographic characteristics of community relays

We surveyed 160 community health workers. Three out of four community relays were men and aged 25 to 45 (80%). The Sahouè ethnic group was predominant (56.9%), and the majority practiced a Christian (46.3%) or traditional (48.8%) religion.

Most RCs had a secondary education (71.3%) and lived in a common-law relationship (78.1%). Professionally, they were mainly artisans (30.6%) or retailers (22.6%). Almost all (97.5%) had resided in their village or neighborhood for more than 3 years (Table 1).

**Table 1:** Sociodemographic characteristics community relays in the Comè, Bopa, Grand Popo and Houéyogbé health zone in Benin in 2024.

	Workforce (*n* = 160)	%
Sex		
Female	46	28.8
Male	114	71.3
Age (year)		
25–35	69	43.1
35–45	59	36.9
45–55	23	14.4
55 years and over	9	5.6
Ethnic group		
Adja/Fon	8	5.0
Kotafon	5	3.1
Mina	15	9.4
Pedah	10	6.3
Sahouè	91	56.9
Watchi	21	13.1
Xwla	10	6.3
Religion		
None	5	3.1
Christian	77	48.2
Traditional	78	48.8
Educational level		
Primary	27	16.9
Secondary	114	71.3
University	19	11.9
Marital status		
Bachelor	5	3.1
Bride	30	18.8
Free union	125	78.1
Occupation		
Craftsman	49	30.6
Farmer	34	21.2
Other	17	10.6
Housewife	16	10.0
Fisherman	8	5.0
Reseller	36	22.6
Place of residence		
This village/neighborhood	156	97.5
Neighboring village/district	4	2.5
Time at place of residence		
< 3 years	7	4.4
> 3 years	153	95.6

### Characteristics related to the task and benefits of the status of community relays

Almost all RCs (96.9%) had at least 4 years of experience and 81.9% had already used an Android tablet or smartphone. In addition, 85% had received training on the activity package, and 88.1% were trained on water, hygiene and sanitation; a Qualified Community Health Agent (QCHA) followed 91.3%. In addition, 66.3% had received in-service training, and 80% participated in at least three group follow-up sessions (Table 2).

**Table 2:** Distribution of community relays according to their professional characteristics and the advantages linked to their status, health zone Comè, Bopa, Grand Popo and Houéyogbé, 2024.

	Workforce (*n* = 160)	%
Old and new status		
Yes	155	96.9
No	5	3.1
Android or tablet layout		
Yes	25	15.6
Yes view	131	81.9
No	4	2.5
Training on the activity package		
Yes	136	85.0
No	24	15.0
Water, hygiene and sanitation training		
Yes	141	88.1
No	19	11.9
ASCQ monitoring		
Yes	146	91.3
No	14	8.8
Number of group follow-up sessions		
Zero	20	12.5
A	7	4.4
Two	5	3.1
Three	88	55.0
Four and more	40	25.0
Continuing education received		
No	54	33.8
Yes	106	66.3
Free consultation at CS		
Yes	7	4.4
No	153	95.6
Official presentation		
Yes	109	68.1
No	51	31.9
Benefits and bonuses		
Yes	135	84.4
No	25	15.6
Involvement in mass vaccination campaigns		
Yes	152	95.0
No	8	5.0

Only 4.4% benefited from free consultations at the health center. However, 84.4% received bonuses and 95% participated in vaccination campaigns.

### Knowledge of community relays on water, hygiene and sanitation

The results on the knowledge of the RCs show that 50% knew the types of activities carried out, but only 47.5% mastered their roles and responsibilities. Similarly, 93.1% organized communication for behavior change sessions, and 81.3% carried out household visits. Nevertheless, 31.2% had good knowledge while 68.8% had insufficient knowledge on water, hygiene and sanitation (Table 3).

**Table 3:** Distribution of community relays according to their knowledge of their roles and responsibilities.

	Workforce (*n* = 160)	%
Knowledge of the types of activities carried out		
Yes	80	50.0
No	80	50.0
Knowledge of roles and responsibilities		
Yes	76	47.5
No	84	52.5
Organization of a communication session for change		
Yes	149	93.1
No	11	6.9
Organization of visits to 200 households		
Yes	130	81.3
No	30	18.8
Level of knowledge of RCs		
Good knowledge	50	31.2
Insufficient knowledge	110	68.8

### Factors influencing knowledge of community health workers

Several factors influenced knowledge of RCs, including: Ethnicity (OR = 0.3; 95% CI = [0.1–0.9]) and marital status (OR = 10.0; 95% CI = [1.3–77.7]) significantly influenced knowledge of RCs. RCs who received training on the activity package (OR = 3.3; 95% CI = [1.7–10.0]) and those followed by an ASCQ (OR = 10.2; 95% CI = [2.5–40.6]) were more likely to have good knowledge. The number of group follow-up sessions was a key factor: those who attended at least three sessions (OR = 10.0; 95% CI = [5.0–48.9]) were more likely to have good knowledge. The official presentation of RC at the locality (OR = 2.0; 95% CI = [1.1–5.0]), bonuses (OR = 2.0; 95% CI = [1.5–10.1]) and involvement in vaccination campaigns (OR = 10.0; 95% CI = [2.0–50.0]) are associated with good knowledge.

In the multivariate analysis, RCs who participated in two group follow-up sessions were 23.9 times more likely to have good knowledge about water, hygiene and sanitation compared to those who did not participate in any session (OR = 23.9; 95% CI = [5.31–107.72]) than the others (Table 4).

**Table 4:** Factors associated with knowledge of community relays in the Comè, Bopa, Grand Popo and Houéyogbé health zones in Benin in 2024.

Variables	OR	95% CI		*p*-value
Ethnic group				0.029
Adja/Fon	0.3	0.1	1.1	
Kotafon	0.2	0.03	1.1	
Mina	0.3	0.1	0.9	
Pedah	0.4	0.1	1.7	
Sahouè	1	-	-	
Watchi	0.4	0.1	0.9	
Xwla	0.4	0.1	0.9	
Marital status				0.030
Bachelor	1			
Bride	10.0	1.3	77.7	
Free union	3.3	0.5	10.0	
Old and new status				0.017
Yes	3.3	1.7	10.0	
No	1			
Training on the activity package				0.002
Yes	3.3	1.7	10.0	
No	1			
Water, hygiene and sanitation training				0.000
Yes	5.1	2.3	10.2	
No	1			
ASCQ monitoring				0.000
Yes	10.2	2.5	40.6	
No	1			
Number of group follow-up sessions				0.000
Zero	1			
one	0.0	0.0	4.4	
Two	5.0	0.7	48.9	
Three	10.1	5.0	20.2	
Four and more	10.0	5.0	45.3	
Official presentation				0.026
Yes	2.0	1.1	5.0	
No	1			
Benefits and bonuses				0.000
Yes	2.0	1.5	10.1	
No	1			
Involvement in mass vaccination campaigns				0.002
Yes	10.0	2.0	50.0	
No	1			

## Discussion

The results of this study show that 31.2% of RCs had good knowledge. Our findings highlight that CHW knowledge is strongly influenced by programmatic factors such as training, supervision, and recognition, rather than by individual characteristics such as age, sex, or education level. Specifically, CHWs who received refresher training, were supervised by a qualified agent, and participated in multiple group follow-up sessions had significantly better knowledge. Incentives such as official recognition and bonuses also played an important role. These results suggest that institutional support mechanisms are decisive for CHW performance. This aligns with evidence from other West African studies, which found that capacity building, continuous supervision, and motivational strategies are essential to ensure effective community health promotion in WASH (Table 5).

**Table 5:** Final model of the multivariate analysis resulting from logistic regression in the Comè, Bopa, Grand Popo and Houéyogbé health zones in Benin in 2024.

Variables	ORa	95% CI		*p*-value
Number of group follow-up sessions				
Zero	1			
one	6.0	0.7	48.9	0.112
Two	23.9	5.3	107.7	0.000
Three	9.9	2.8	34.8	0.000
Four and more	–			0.99

Nearly three-quarters of the CHWs were men and were predominantly between the ages of 25 and 45. The male predominance could be explained by sociocultural norms that favor men’s involvement in community roles. Studies conducted in Kenya and Côte d’Ivoire had observed a male predominance among community health workers, suggesting that this could be linked to the perception of community work as a responsibility requiring frequent travel and increased social interaction (12, 13). In contrast, a study from Ethiopia have reported a predominance of female CHWs, often explained by cultural acceptance of women in maternal and child health roles, and the perception that women can more effectively reach households for health promotion activities (14). This contrast highlights the influence of cultural norms and gender roles on CHW recruitment patterns.

The majority of RCs had reached a secondary level, which should theoretically make it easier for them to acquire knowledge about water, hygiene and sanitation. However, the high proportion of RCs with insufficient knowledge (68.8%) highlights that the level of education does not automatically guarantee a good understanding of health issues. A study conducted in Burkina Faso also showed that knowledge about hygiene and sanitation did not depend solely on the educational level, but also on the quality of the training received (15). Similarly, evidence from Ghana indicates that targeted, practical training adapted to local contexts significantly improves WASH knowledge compared to general health education, regardless of formal education level (16).

Training plays a key role in knowledge acquisition. Our results show that community workers who received training on the activity package (OR = 3.3; [1.7–10.0]) are more likely to have good knowledge. Studies in Côte d’Ivoire have shown that training adapted to local realities significantly improves the practices of community workers (15, 17).

Our results indicate that follow-up by the Qualified Community Health Worker and participation in-group follow-up sessions are determining factors. An analysis conducted in Uganda also highlighted the positive impact of monitoring community workers on improving their knowledge and commitment (18). Community workers who receive incentives and participate in mass vaccination campaigns have better knowledge of water, hygiene and sanitation practices; official recognition and financial benefits are key elements to motivate and retain community workers (12).

The results of our study are consistent with those of other research conducted in West Africa. An assessment conducted around Lake Nokoué in Benin showed that 56.79% of respondents had never received hygiene training and 84.29% did not have access to latrines (19, 20). Furthermore, a study conducted in Côte d’Ivoire highlighted the importance of continuing education to ensure a sustainable impact of health interventions (15). These findings suggest that national health policies should institutionalize periodic WASH-focused training and ensure infrastructure improvements, especially in underserved areas, to maximize CHWs’ impact.

Self -reported knowledge by RCs may also introduce social desirability bias. Longitudinal research would be needed to better understand the evolution of knowledge and identify predictors of good understanding of hygiene and sanitation practices (21, 22). Despite a relatively high level of education, a significant proportion of RCs have insufficient knowledge of good water, hygiene and sanitation practices. Training, regular monitoring and adequate incentives could improve this situation, making RCs more effective in their community awareness-raising role. In resource-constrained settings, strengthening CHWs’ WASH competencies is a strategic priority for reducing preventable diseases. Policies should therefore align training, supervision, and incentive structures to ensure sustainability and scalability of interventions.

## Conclusion

Community health workers (CHW) play a central role in advancing public health, particularly in water, hygiene, and sanitation (WASH). However, insufficient knowledge among many CHW limits their impact. To address this, ongoing training, regular monitoring, and motivation strategies (e.g., official recognition or incentives) are essential to enhance their effectiveness. These measures would strengthen public health outcomes in communities. Longitudinal studies are further needed to assess CHW’ evolving knowledge and their direct impact on improving community hygiene and sanitation practices.

## Data Availability

The datasets presented in this study can be found in online repositories. The names of the repository/repositories and accession number(s) can be found in the article/supplementary material.
